# Microscopic Colitis: Pathogenesis and Diagnosis

**DOI:** 10.3390/jcm12134442

**Published:** 2023-07-01

**Authors:** Busara Songtanin, Jason N. Chen, Kenneth Nugent

**Affiliations:** 1 Department of Internal Medicine, Texas Tech University Health Sciences Center, Lubbock, TX 79430, USA; busara.songtanin@ttuhsc.edu; 2 School of Medicine, Texas Tech University Health Sciences Center, Lubbock, TX 79430, USA; jason.chen@ttuhsc.edu

**Keywords:** microscopic colitis, collagenous colitis, lymphocytic colitis, chronic diarrhea, pathogenesis, diagnostic tests

## Abstract

**Simple Summary:**

Patients with microscopic colitis have chronic watery diarrhea. The cause of this disorder is uncertain. Some patients appear to have a genetic predisposition, some patients have changes in the bacterial flora in their colon, and some patients have increased amounts of bile acid in their colon. These patients often have autoimmune disorders, such as celiac disease. No laboratory tests can establish this diagnosis. These patients must undergo colonoscopy with biopsy. The pathologist usually classifies these patients as lymphocytic colitis with increased numbers of lymphocytes in the mucosa or collagenous colitis with an increase in collagen deposition in the submucosa. Budesonide is the recommended initial treatment, which is a poorly absorbed oral corticosteroid medication. Some patients improve when treated with medications that bind bile acids.

**Abstract:**

Microscopic colitis is a type of inflammatory bowel disease and is classified as either collagenous colitis or lymphocytic colitis. The typical presentation is chronic watery diarrhea. The disease occurs more frequently in women aged 60–65 years and is increasing in incidence. The pathophysiology of microscopic colitis remains poorly understood and has not been well-described with possible several pathogeneses. To date, the diagnosis of microscopic colitis depends on histological tissue obtained during colonoscopy. Other non-invasive biomarkers, such as inflammatory markers and fecal biomarkers, have been studied in microscopic colitis, but the results remains inconclusive. The approach to chronic diarrhea is important and being able to differentiate chronic diarrhea in patients with microscopic colitis from other diseases, such as inflammatory bowel disease, functional diarrhea, and malignancy, by using non-invasive biomarkers would facilitate patient management. The management of microscopic colitis should be based on each individual’s underlying pathogenesis and involves budesonide, bile acid sequestrants, or immunosuppressive drugs in refractory cases. Cigarette smoking and certain medications, especially proton pump inhibitors, should be eliminated, when possible, after the diagnosis is made.

## 1. Microscopic Colitis

Microscopic colitis is an inflammatory bowel disease possibly caused by a chronic immune-mediated process. The incidence rate of microscopic colitis is estimated to be 11.4 per 100,000 person-years [1]. The mean age at diagnosis is 60–65 years with a female-to-male ratio as high as 9:1 [2]. It is a common cause of chronic watery non-bloody diarrhea. The disease is classified into collagenous colitis and lymphocytic colitis based on histologic findings. Collagenous colitis was first described in 1976 [3], and lymphocytic colitis was first described in 1989 [4]. Microscopic colitis is a common cause of chronic watery diarrhea and has a clinicopathological triad of chronic watery non-bloody diarrhea, normal mucosal appearance on colonoscopy, and distinct characteristic histopathology that meet the diagnostic criteria of collagenous colitis or lymphocytic colitis [5]. Microscopic colitis affects all segments of the colon, excluding the rectum; however, the disease process does not affect the colon uniformly [6]. Current literature suggests that the histological findings may be patchy and not continuous throughout the colon and that it is the most severe in the proximal part of the colon [7]. Furthermore, biopsies taken from the rectum have the highest rates of false negative results and are not recommended [8]. This review article considers the pathogenesis of microscopic colitis and diagnostic tests relevant to the pathogenesis and briefly discusses treatment.

## 2. Pathogenesis of Microscopic Colitis

To date, the pathogenesis of microscopic colitis remains unknown and is likely complex and multifactorial [1,9]. The possible mechanisms include intraluminal factors, such as abnormal microbiota, genetic predisposition, bile acids, and autoimmunity, which could trigger chronic inflammation [9,10,11]. This section of the review will focus on four pathogenetic processes: genetic predisposition, microbiome alteration, immunological processes, and bile acid malabsorption (See the graphical abstract).

### 2.1. Genetic Predisposition

The role that genetics have in the pathogenesis of microscopic colitis is not completely understood. It has been proposed that microscopic colitis is the result of an abnormal immune response to luminal antigens in genetically predisposed individuals [10]. However, there have been very few genome-wide studies that explored the associations between genes and microscopic colitis. Westerlind et al. used immunochip and targeted single nucleotide polymorphism (SNP) genotype data from 314 collagenous colitis patients, 122 lymphocytic colitis patients, and 4299 controls to analyze possible associations. This study found that human leukocyte antigen (HLA) 8.1 haplotype variants were associated with an increased risk for collagenous colitis [12]. Using the same technique, the authors found no associations for an increased risk for lymphocytic colitis [13]. Genetic similarities were also found between inflammatory bowel disease (IBD) and collagenous colitis [12].

These results have been confirmed by two more recent studies. Green et al. used UK Biobank SNP genotypes of white Europeans, which included 483 patients with microscopic colitis and 450,616 controls. This study confirmed the results of Westerlind et al. and found that the HLA 8.1 haplotype SNP was associated with microscopic colitis [14]. The authors also found that white Europeans with microscopic colitis had an eight times higher risk of celiac disease and a 12 times higher risk of IBD than did controls. Stahl et al. further supported these findings by combining retrospective data and added cases for a total of 804 collagenous colitis DNA samples. These authors reported similar results of an increased risk for collagenous colitis in those with HLA alleles related to the ancestral haplotype 8.1. They also found genetic overlap between collagenous colitis, celiac disease, Crohn’s disease, and ulcerative colitis [15].

Jarnerot reported five families with familial microscopic colitis proven by histological tissue in sisters [16]. This study suggested that there is no association with environmental factors in the pathogenesis of microscopic colitis since the two sisters in one family did not live in the same country. This report included the following relationships: family A: sister 1 collagenous colitis–sister 2 collagenous colitis; family B: sister 1 collagenous colitis–sister 2 microscopic colitis (but another sister without microscopic colitis); family C: sister 1 collagenous colitis–sister 2 collagenous colitis; family D: sister 1 collagenous colitis–sister 2 collagenous colitis; family E: sister 1 collagenous colitis–sister 2 lymphocytic colitis. This study suggests that there may be a genetic basis for some cases with microscopic colitis and makes family history useful information during the evaluation of patients with chronic diarrhea.

### 2.2. Gut Microbiome

The gut microbiome is another factor that may have implications in the pathogenesis of microscopic colitis. Early studies have noted the development of microscopic colitis after infections from *Clostridium difficile* and *Yersinia* [17,18]. These findings, combined with reports that diversion of fecal material reduces mucosal inflammation in collagenous colitis [16,19], support the proposal that changes in the microbiota and, thus, the microbiome, affect microscopic colitis development. This idea is supported by a fecal study that compared the microbiota of 10 patients with collagenous colitis with 10 healthy controls, both before and after treatment with budesonide [20]. At baseline, the diversity of the microbiota was less in the patients with collagenous colitis compared to the healthy individuals. Treatment with budesonide led to increased diversity in the collagenous colitis microbiome, which brought it closer to the composition found in the healthy controls. This suggests that the microbiome may contribute to the development of microscopic colitis and that targeting the microbiome could be a potential therapeutic approach. Similar findings were reported by Morgan et al., who collected fecal samples from 20 patients with microscopic colitis during both active and remission phases of this disorder, from 20 patients with functional diarrhea (i.e., non-organic) and from 20 healthy control subjects [21]. There was a significant decrease in variability in the microbiota during the active phase compared to the remission phase. Although not statistically significant, there was also lower diversity in active microscopic colitis than in the healthy controls. In addition, microbial dysbiosis was significantly higher in an active phase microscopic colitis compared to patients in a remission phase, healthy controls, and patients with functional diarrhea. Analysis of the microbiota composition found a higher proportion of *Haemophilus parainfluenzae*, *Veillonella parvula*, and *Veillonella* species in patients with microscopic colitis than in healthy controls, and fewer *Alistipes putredinis*. This may be a significant finding due to the protective anti-inflammatory implications of *Alistipes* species [22]. 

Lower diversity in the microbiota was also reported in studies by Sun et al. and Hertz et al. [23,24]. Sun and colleagues compared microbiota composition from biopsies of the ascending and descending colons from 52 microscopic colitis patients and 153 healthy controls. They found increased *Proteobacteria* in microscopic colitis and increased *Collinsella* in healthy controls. These changes occurred in both the ascending and descending colon. Hertz and colleagues reported increased *Prevotella* enrichment in microscopic colitis gut microbiota from 15 fecal samples compared to 21 healthy control fecal samples [21].

There have been additional smaller-scale studies conducted to examine the microbiota composition in patients with microscopic colitis. Gustafsson et al. studied the colon microbiota of two women with collagenous colitis and found a predominance of *Bacteroides* and *Firmicutes* species similar to that of a healthy colon [25]. However, it was found that there was a higher proportion of *Bacteroides* (47.0% and 31.1% of clones in these two patients) compared to previously published data on healthy guts (3.4–24.4% of clones) [26,27,28]. In addition, *Clostridium clostridioforme* was found in both patients, which has been historically associated with a variety of infections [29]. *Akkermansia mucinciphila*, a protective species known to increase the turnover of the mucin layer that helps protect against toxic material [30], was not found in these two women. Fischer supported these results using DNA sequencing to compare the fecal microbiota from 10 female patients with microscopic colitis with the microbiota from seven healthy control women. As in the study by Gustafsson et al., patients with microscopic colitis had a significant reduction in *Akkermansia* species compared to the healthy control subjects [31]. From colon tissue samples of 20 microscopic colitis patients and 20 controls, Millien et al. found that patients had an increased number of the proinflammatory family *Desulfovibrionales* and had fewer numbers of the *Coriobacteriaceae* family that is abundant in a healthy gut [32]. Although these small-scale studies have emphasized the importance of studying microbiota, larger-scale longitudinal studies are needed to better understand the role that the microbiome has on the pathophysiology of *Desulfovibrionales* and other inflammatory microbes.

Studies on the colonic microbiome in patients with microscopic colitis could provide the basis for diagnostic testing if there are consistent changes and if treatment changed the microbiome and improved symptoms.

### 2.3. Autoimmune-Related Colonic Injury

Autoimmunity is a possible pathophysiological mechanism in microscopic colitis. A nationwide case-controlled study performed in Denmark recruited 15,597 microscopic colitis patients and 155,910 controls and reported a significant correlation of autoimmune disease in microscopic colitis with an adjusted odds ratio (OR) of 2.9 (95% CI: 2.0–2.2). The highest ORs were in patients with celiac disease (OR 10.2; 95% CI: 8.2–12.6), Crohn’s disease (OR 2.5; 95% CI; 2.1–2.9), and ulcerative colitis (OR: 6.7; 95% CI; 6.2–7.3) [33]. The same study also demonstrated an increase in the prevalence of autoimmune disease in collagenous colitis compared to lymphocytic colitis (OR 2.4; 95% CI, 2.3–2.5) and (OR 1.9; 95% CI: 1.7–2.0), respectively. However, the positive correlation of IBD and microscopic colitis in this study might have had a misclassification bias due to overlapping histologic and endoscopic findings in these two groups combined with a lack of clinical data. The association was higher in younger patients aged 18–49 compared to older patients aged 50–59. Another retrospective study review of 103 patients with microscopic colitis reported that 40 (39%) of patients had an underlying autoimmune disease with no difference between patients with collagenous colitis and lymphocytic colitis [34]. Hashimoto thyroiditis was the most prevalent autoimmune disease in fourteen patients (35%), followed by rheumatoid arthritis in seven (17.5%), and Sjogren’s syndrome in seven (17.5%).

Studies on the immunopathological mechanisms in microscopic colitis are limited to small case series. Göranzon studied the characteristics of lymphocytes in the colonic biopsy tissue samples from 23 patients with microscopic colitis and 17 control patients. In the microscopic colitis group (both collagenous colitis and lymphocytic colitis), the biopsies showed a significant increase in the number of CD8^+^ lymphocytes in the epithelium and lamina propria and a decreased number of CD4^+^ lymphocytes in the lamina propria [35]. This study reported an increase in CD3^+^ lymphocytes in the epithelium in collagenous colitis and lymphocytic colitis compared to the control patients, with a significant increase in CD3^+^ in the lymphocytic colitis group. This study also found an increase in CD3^+^ and CD 20 (B lymphocytes) in microscopic colitis patients. It should be noted that the controls in this study were not healthy controls, and some likely had IBS as the study recruited patients who had a normal colonoscopy but had presented with intestinal bleeding and changes in bowel habits.

Sandler et al. studied the association between clinical symptoms in patients with microscopic colitis and lymphocyte infiltration in the epithelium and lamina propria in 97 microscopic colitis patients and 165 controls [36]. There were increased numbers of CD8+ lymphocytes in both the epithelium and lamina propria of the patients with microscopic colitis, but there was no associations between diarrhea symptoms and T-cell infiltration in the patients with microscopic colitis. This study indicates that patients with microscopic colitis with more frequent stools do not necessarily have more T-cell infiltration in the colonic mucosa.

An autoimmune disorder might explain the development of microscopic colitis in some younger patients and the beneficial effects of treatment with an oral corticosteroid medication. In addition, developing tests for antibodies against colonic antigens could lead to a simple diagnostic test. For example, Kuwada et al. demonstrated that the majority of patients with ulcerative colitis had autoantibodies against integrin αvβ6 and suggested that this antibody might serve as a potential diagnostic marker with high sensitivity and specificity in these patients [37]. 

### 2.4. Bile Acid Malabsorption

The association between bile acid malabsorption and microscopic colitis remains poorly understood and is complicated by the complex physiology and metabolism of bile acids in the intestinal tract [38,39,40]. In addition, there are several receptors in the intestinal tract that bind bile acids, have important physiologic effects, and complicate any analysis of bile acid physiology in the colon. These acids have both beneficial physiologic effects that help maintain colonic health and support nutrition and can have adverse effects that contribute to the development of diarrhea. Bile acids help maintain intestinal epithelial barrier function and contribute to the formation of tight junctions. They have antibacterial effects and help maintain a “healthy” intestinal microbiome, and they support beneficial immune responses in the colon. There are at least seven receptors in colonic tissue that bind bile acids and have physiologic effects [38]. For example, the Farnesoid X-receptor (FXR) decreases the synthesis of bile acids in the liver, and it also regulates innate and adaptive immunity. The binding of bile acids to this receptor decreases the secretion of tumor necrosis factor alpha, and this limits or controls the development of inflammation. The density of this receptor is increased by luminal bile acids and decreased by inflammation; therefore, the number of receptors will depend on a complex set of conditions and may not be predictable. The adverse effects of bile acids include increased secretion of chloride, decreased absorption of fluid, and increased colonic motility which can contribute to chronic diarrhea.

The development of diarrhea associated with bile acid malabsorption should reflect either increased synthesis of bile acids or decreased absorption in the terminal ileum. In addition, the metabolism of primary bile acids into secondary bile acids depends on the bacteria present in the colon, and alterations in the bacterial flora could influence the composition of bile acids in the colon. In some patients, bile acids could have a primary effect and cause microscopic colitis. Alternatively, these acids could have a secondary effect and increase diarrhea in patients with established microscopic colitis. The number of bile acid receptors and the complexity of the metabolism of bile acids in the intestinal tract make the analysis of any effect on bile acids on colonic disease quite difficult. 

The pathogenesis of bile acid malabsorption in patients with microscopic colitis likely has several mechanisms. There is evidence of villous atrophy and inflammation in the ileum in some patients with microscopic colitis, and this could lead to bile acid malabsorption and increased concentration of bile acids in the colon [41,42,43]. Padmanabhan et al. studied the histological features of the terminal ileum in thirty-two patients with lymphocytic and collagenous colitis [44]. The mean number of lymphocytes per 100 epithelial cells was significantly higher in patients than in controls. In lymphocytic colitis the mean number was 22.3 ± 10.2, in collagenous colitis the mean number was 16.7 ± 6.1, and in control biopsies the mean number was 8.2 ± 2.9. Fourteen patients out of eighteen with lymphocytic colitis had a mean number of intraepithelial lymphocytes greater than two standard deviations above the control mean. Seven out of fourteen patients with collagenous colitis had a mean number greater than two standard deviations above the control. The lymphocytes in these patients were T cells. In some patients the surface of the ileum was abnormal; four had villous atrophy, five had epithelial damage with loss of mucin, and three had an increase in subepithelial collagen. This study demonstrates that patients with microscopic colitis can have abnormal mucosa in the terminal ileum. Involvement of the ileum in these patients may reflect “spread” of the disease from the colon back into the ileum. Alternatively, some of these patients may have undiagnosed celiac disease, which involves the ileum. These patients could have decreased bile absorption of the terminal ileum and therefore increased bile acids in the colon, which could influence the development and persistence of diarrhea.

A few studies have used ^75^SeHCAT, a highly sensitive and specific test for bile acid malabsorption, to determine the prevalence of bile acid malabsorption in patients with microscopic colitis. However, the hypothesis that bile acid malabsorption is associated with microscopic colitis is complicated by several other studies that show that microscopic colitis patients with normal ^75^SeHCAT results also responded to cholestyramine [45,46,47]. This result might suggest that bile acids can have an important effect in patients with a “vulnerable” mucosa even at low levels. The following studies provide more information on the association of microscopic colitis with bile acid malabsorption and/or treatment of patients with microscopic colitis with bile acid sequestrants.

Ung et al. comprehensively evaluated twenty-seven patients with collagenous colitis. Twelve of these patients had bile acid malabsorption based on the ^75^SeCHCAT test [45]. Stool frequency was higher in patients with colitis and bile acid malabsorption than in patients without bile acid malabsorption. Eleven of these patients had at least one autoimmune disease. All patients received a bile acid binder. Rapid improvement occurred in twenty-one of twenty-seven patients. Eleven of the twelve patients with bile acid malabsorption had a rapid response; ten of the fifteen patients without bile acid malabsorption had a rapid response. These authors note that the etiology of the bile acid malabsorption of these patients is unclear, and that the effect of bile acids on colonic function and the production of diarrhea is also unclear. Presumably, the bile acids could cause a low-grade injury to the mucosa and/or alter electrolyte absorption.

Ung and coinvestigators did a similar study on patients with lymphocytic colitis [46]. Only two out of twenty-three patients had a low retention of ^75^SeCHAT. Six patients out of thirteen treated with a bile acid binder responded to treatment. During follow-up, two patients developed collagenous colitis. Both studies indicate that patients with bile acid malabsorption associated with microscopic colitis can have a good clinical response to bile acid sequestrants. In addition, some patients without biochemical evidence of bile acid malabsorption also can respond to bile sequestrants. Fernandez et al. reported that 43.1% of their patients with microscopic colitis also had bile acid malabsorption [7]. In their study, malabsorption was more frequent in lymphocytic colitis patients than collagenous colitis patients, affecting 60% compared to 27%, respectively.

Northcutt analyzed the effect of bile acid sequestrant therapy in seventy-nine patients with microscopic colitis following a registry in United States [48]. Forty-six of these seventy-nine patients (58.2%) had a response to this therapy. In forty-six budesonide-dependent patients, twenty-three patients had a response to treatment. Several patients in this registry also had an autoimmune disease; five patients had celiac disease, eleven patients had hypothyroidism, and six patients had one of the following disorders, including diabetes, psoriasis, ulcerative colitis, lichen planus, and systemic lupus. These authors noted that bile acid malabsorption is present in patients with microscopic colitis in 24% to 44% based on ^75^SeHCAT retention. However, they noted that some patients without bile acid malabsorption based on this test also responded to bile acid sequestrant therapy. Possible mechanisms include the fact that bile acid sequestrant therapy binds deoxycholic acid, which increases colonic secretion of water and electrolytes. Bile acid sequestrants also bind chenodeoxycholic acid, which has a prokinetic effect through increased colonic contractions. Budesonide may have its clinical effect explained in part by the fact that there is a reduced bile acid load in the colon in patients on budesonide.

Gurbuz et al. reported a 44-year-old man who developed lymphocytic colitis diagnosed by biopsy after a cholecystectomy [49]. The patient’s diarrhea symptoms were controlled with cholestyramine but relapsed after initially stopping the medication. His symptoms eventually resolved after continuing the cholestyramine again. This case indicates that surgical history is important in some patients with microscopic colitis. However, other studies showed that microscopic colitis is not usually associated with cholecystectomies or appendectomies [50].

The European guidelines for microscopic colitis recommend testing for bile acid malabsorption as part of a routine diagnostic work-up in patients with microscopic colitis who do not respond to budesonide treatment, but the guideline did not recommend testing for bile acid diarrhea as part of a routine diagnostic workup in microscopic colitis, even though MC and bile acid malabsorption can present with similar symptoms. Some patients with MC with both bile acid malabsorption and without bile acid malabsorption respond to bile acid sequestrants [1].

In summary, these studies indicate that bile acids may have a primary or secondary effect in microscopic colitis. Multiple factors, including the bile acid load in the colonic lumen, bile acid metabolism, and complex physiologic effects mediated by several receptors, make this association very difficult to study. The simplest approach for clinicians managing these patients would involve the measurement of bile acid malabsorption and the use of bile acid sequestrants in empiric trials with these patients.

## 3. Microscopic Colitis and the Differential Diagnosis of Chronic Diarrhea

**Figure 1:**
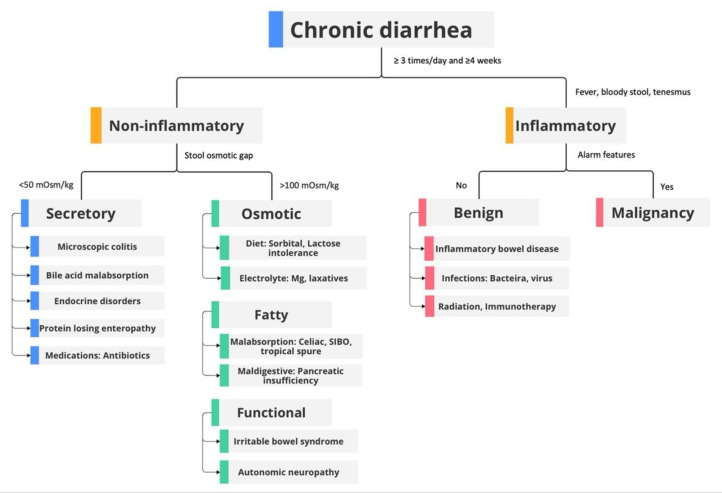
Diagnostic approach to chronic diarrhea. Adapted from https://clinicalproblemsolving.com/dx-schema-chronic-diarrhea-2/, accessed on 1 June 2023. SIBO, small intestinal bacterial overgrowth.

Chronic diarrhea is defined as loose watery stools that occur three or more times within one day and last for four or more weeks [51]. This condition affects 5% of the total population at any given point in time [51]. Chronic diarrhea can be classified into several categories: inflammatory and non-inflammatory, secretory and osmotic, or organic and functional diarrhea. Organic diarrhea is the group of medical conditions that are associated with physiologic, structural, or biochemical abnormalities whereas functional diarrhea is characterized by conditions that are not explained by structural or biochemical abnormalities (Figure 1) [52]. The differential diagnosis of chronic diarrhea is broad and includes celiac disease, inflammatory bowel disease, irritable bowel syndrome, colorectal cancer, infection, bile acid malabsorption, drug-induced, etc. [53]. A detailed history should be obtained when evaluating patients with chronic diarrhea. Patients with any alarm features should raise concerns for organic diarrhea. According to the American Gastroenterological Association (AGA), “alarm symptoms” include weight loss, anemia, and hypoalbuminemia [54]. Other alarm features that have been described include persistent blood in stool, change in bowel habits [55], nocturnal pain or diarrhea, and a first-degree relative with inflammatory bowel disease or colorectal cancer. History regarding recent travel, recent antibiotics used (risk for *Clostridium difficile* infection), medications, dietary changes, and prior surgeries should be obtained. Patients with underlying autoimmune diseases, such as type two diabetes mellitus, thyroid disorder, iron deficiency anemia, and infertility, should raise suspicion for celiac disease [53]. Among functional gastrointestinal disorders, irritable bowel syndrome (IBS) is the most prevalent [56]. Irritable bowel syndrome-diarrheal type can be diagnosed by clinical symptoms with ROME IV criteria and symptoms usually improves at night and with fasting [56]. The AGA recommends the use of either fecal calprotectin or lactoferrin and recommends against the use of erythrocyte sedimentation rate (ESR) and C-reactive protein (CRP) in screening for IBD [54]. Laboratory tests recommended for initial workup include stool tests for *Clostridioides difficile* and routine stool cultures (*Salmonella, Shigella, Campylobacter, Yersinia*, *Escherichia coli O157:H7)*. Stool tests for ova and parasites (three samples) should also be performed, particularly if the patient has risk factors, such as recent travel to endemic areas. A complete blood count, electrolytes, and albumin should be obtained since patients with microscopic colitis may have mild anemia and, in rare cases, a protein-losing enteropathy. 

Secretory diarrhea can be differentiated from osmotic diarrhea by history. Patients with secretory diarrhea often have nocturnal diarrhea and persistent symptoms despite fasting, whereas osmotic diarrhea symptoms improve with fasting. These conditions can also be differentiated by calculating the fecal osmotic gap (equation 290 − 2× (stool Na^+^ + stool K^+^)). In secretory diarrhea, the fecal osmotic gap is <50 mOsm/kg, whereas in osmotic diarrhea this gap is >100 mOsm/kg. Patients with fat malabsorption diarrhea, e.g., celiac disease, amyloidosis, small intestinal bacterial overgrowth, and pancreatic exocrine insufficiency, will have increased fecal fat and high stool volume [53].

Therefore, it is important to differentiate microscopic colitis from other conditions which present with secretory diarrhea. The mechanism for secretory diarrhea in collagenous colitis can be explained by a decreased Cl/HCO_3_ exchange rate and increased chloride secretion; the underlying mechanism of diarrhea in lymphocytic colitis is a decrease of active sodium absorption [57]. In patients whose history and laboratory demonstrated secretory diarrhea, the clinician must exclude medication-induced, bile acid malabsorption, endocrine disorders, and postsurgical bowel resection [53]. Given its normal mucosal appearance during endoscopic evaluation, the current diagnostic guideline relies on histological tissue obtained from colonoscopy. However, many studies have proposed laboratory markers and fecal biomarkers to help with the diagnosis of microscopic colitis. In the next section, we will focus on the multiple diagnostic methods used in evaluating microscopic colitis.

See Figure 1 for an overview of the diagnostic approach in patients with chronic diarrhea.

## 4. Standard Diagnosis: Colonoscopy

Microscopic colitis is a chronic inflammatory disease of the colon that is increasing in incidence [58]. At present, there is no specific biomarker for microscopic colitis, and the diagnosis requires histopathological tissue obtained during a colonoscopy, which is an invasive and costly procedure. The European guideline recommends screening for celiac disease in patients with microscopic colitis [1].

Kane et al. did a cross-sectional study of 540 cases of microscopic colitis and reported 89 cases (16.5%) had macroscopic findings [59]. Macroscopic changes from the most common to the least common include erythema, petechiae, edema or congestion, and ulceration. Patients with collagenous colitis had the highest prevalence of macroscopic changes (19.2%) in comparison to patients with lymphocytic colitis (14.8%) and microscopic colitis not otherwise specified (6.5%) [59].

Since the colon usually appears normal during colonoscopy, studies have tried to determine the best biopsy location and the number of biopsies needed during colonoscopy to optimize the diagnosis of microscopic colitis due to potential patchy distribution of the disease [60]. Currently, the European Guideline strongly recommends that ileocolonoscopy should be performed in patients with suspected microscopic colitis and that biopsies should be taken from the right and the left side of the colon [1]. Regarding the number of biopsies, the American Society of Gastrointestinal Endoscopy (ASGE) recommends taking at least eight biopsies during colonoscopy by taking two or more biopsies from the right, transverse, left, and sigmoid colon; however, this guideline has not been validated and is based on expert opinion [60]. A recent systematic review revealed that the tissue obtained from the ascending colon and the descending colon had the highest diagnostic rate and a minimum of six biopsies should be obtained (three from the ascending colon and three from the descending colon) to ensure a high certainty in diagnosing microscopic colitis [61]. Tanaka studied the distribution of collagenous colitis and reported that rectal biopsy should be avoided since the disease is more prominent in the proximal part of the colon [8]. Chapman reported a 20% missed rate of diagnosis of microscopic colitis if the biopsies are not obtained above the rectosigmoid colon [62]. A recent retrospective study of 101 patients with microscopic colitis by Virine reported that biopsies obtained from the ascending and the descending colon are adequate for making a diagnosis of microscopic colitis [63]. The tissue obtained only from flexible sigmoidoscopy had low sensitivity and specificity. The use of sigmoidoscopy in obtaining the tissue biopsy (from the left colon) in cases that have a high suspicion of microscopic colitis still showed lower diagnostic sensitivity compared with a standard colonoscopy with multiple colonic sampling sites [63].

Colonoscopy has several possible disadvantages, such as bleeding, infection, adverse reactions from sedation, and perforation [64]. This review will also consider non-invasive diagnostic approaches to microscopic colitis.

## 5. Histological Diagnosis of Microscopic Colitis

According to European guidelines on microscopic colitis published in 2021 [1], collagenous colitis is characterized by a thickened subepithelial collagenous band exceeding 10 mm. Lymphocytic colitis is defined as an increased number of intraepithelial lymphocytes ≥20 per 100 surface epithelial cells combined with an increased inflammatory infiltrate in lamina propria without a significantly thickened collagenous band (<10 μm). Incomplete microscopic colitis comprises incomplete collagenous colitis (defined by a thickened subepithelial collagenous band >5 μm but <10 μm) and incomplete lymphocytic colitis (defined by >10 intraepithelial lymphocytes but <20 intraepithelial lymphocytes and a normal collagenous band). These criteria apply to hematoxylin and eosin-stained slides. Both types show a mild inflammatory infiltrate in the lamina propria.

## 6. Laboratory Biomarkers

### 6.1. Complete Blood Counts (CBC) and Electrolytes

No recent study has analyzed the utility of complete blood counts in the diagnosis of microscopic colitis. However, one study stated that about 50% of patients with microscopic colitis have mild anemia [65]. Bohr reported that in 44 patients with collagenous colitis the mean concentrations of hemoglobin, platelets, serum albumin, alkaline phosphatase, alanine transaminase, aspartate transaminase, and creatinine were normal [47]. Complete blood counts and electrolyte panels are not useful in diagnosis or screening patients who present with chronic diarrhea for microscopic colitis, but these tests may identify important clinical problems that need attention.

### 6.2. Erythrocyte Sedimentation Rate (ESR) and C-Reactive Protein (CRP)

A study by Abboud in 2011 reported that in sixty-three patients with a measured ESR and thirty-six patients with a measured CRP, ten patients (15%) and eight patients (22%) had increased levels, respectively [66]. There was no correlation between ESR and the number of stools, patient global assessment, and physician global assessment (*p*-values of 0.93, 0.96, and 0.16, respectively). After excluding one patient whose results were outliers, there also was no correlation between CRP and the number of stools, patient global assessment, and physician global assessment (*p*-values of 0.90, 0.44, and 0.41, respectively). Another study conducted in Sweden in 1996 with forty-four patients with microscopic colitis reported that the mean ESR in women and men was 25 mm (reference value 2–15 mm) and 12 mm (reference value 2–10 mm), respectively [47]. 

### 6.3. Other Serology Markers

Patients with microscopic colitis can have associated autoimmune diseases, but no specific autoimmune markers have been identified [67]. Roth found an increased prevalence of anti-nuclear antibodies, anti-*Saccharomyces cerevisiae* immunoglobulin G (IgG) antibodies, anti-thyroid peroxidase antibodies, anti-perinuclear neutrophil cytoplasmic antibodies, and anti-glutamic acid decarboxylase antibodies in patients with microscopic colitis compared to a control group [68]. These data were also supported by Holstein who reported a higher prevalence of anti-nuclear antibodies and anti-*Saccharomyces cerevisiae* IgG antibodies in patients with microscopic colitis than in healthy controls [68,69].

## 7. Fecal Biomarkers

Fecal biomarkers have proven to be useful in diagnosing and monitoring patients with inflammatory bowel diseases. Studies on the utility of fecal biomarkers in microscopic colitis have been increasing. White blood cells often have an important role in intestinal inflammation, and this role has been described in tissue biopsy from microscopic colitis patients. The use of fecal neutrophil enzymes, including fecal calprotectin, fecal lactoferrin, and fecal myeloperoxidase, has been studied in patients with microscopic colitis [70]. Therefore, this review on fecal biomarkers will focus on fecal calprotectin, fecal lactoferrin, and fecal myeloperoxidase, as well as fecal eosinophilic proteins, including eosinophil protein X and eosinophil cationic protein.

Eosinophils and neutrophils possibly contribute to the pathogenesis of microscopic colitis [10]. However, this has not been widely studied. One early case report of a collagenous colitis patient showed prominent eosinophils in the lamina propria on histology [71]. Another study analyzing the histology in microscopic colitis reported that in 75.7%, 57.1%, and 11.1% of collagenous colitis, lymphocytic colitis, and incomplete microscopic colitis cases, respectively, more than 20 eosinophils/high power field were found in the lamina propria [72]. 

### 7.1. Fecal Neutrophils

Neutrophils have an important role in “intestinal homeostasis”, which requires cooperative interactions among the epithelium, intestinal microbiomes, and immune cells [73,74]. During the inflammatory process, monocytes release macrophage-derived chemokines that recruit neutrophils to traverse the vascular endothelium to reach the intestinal lamina propria. This transepithelial migration of neutrophils from the circulation into the intestine is an important inflammatory cascade in the pathogenesis of colitis [73]. This transepithelial migration of neutrophils across the intestinal epithelium releases elastase that disrupts E-cadherin-mediated cell-cell contacts resulting in a loss of epithelial barrier function, which then facilitates bacterial translocation from the intestinal lumen to the intestinal mucosa [73]. 

### 7.2. Fecal Calprotectin 

Calprotectin is a calcium and zinc-binding protein that accounts for 60% of the total soluble proteins in the cytosol fraction of neutrophils and is considered to be neutrophil-specific [75]. When the neutrophils migrate to an inflammatory site at the intestinal lumen, the neutrophil disintegrates following oxygen radical generation and releases the cytosol granules that contain calprotectin [75]. The amount of calprotectin reflects the number of participating neutrophils in this inflammation.

The measurement of fecal calprotectin is a useful marker for gastrointestinal inflammation and can be used to screen for inflammatory versus non-inflammatory gastrointestinal disorders, such as IBS. It is particularly useful in assessing for and monitoring IBD [76]. It has a high negative predictive value, which aids in ruling out disease, and a high sensitivity to guide the need for an endoscopy [77]. A meta-analysis showed a pooled sensitivity and specificity of 88% and 73%, respectively, in screening for IBD [78]. Therefore, this is a useful test in distinguishing IBD from IBS and avoiding unnecessary and expensive procedures, such as endoscopy. False elevation in fecal calprotectin can be found in several conditions, such as infection, neoplasia, non-steroidal anti-inflammatory drug use, proton pump inhibitor use, food allergies, age under five years old, and other inflammatory conditions (diverticulitis, microscopic colitis, celiac disease, gastroesophageal reflux disease, cirrhosis) [76]. Given the disease character with lymphocytic infiltration in microscopic colitis, using fecal neutrophils might not reflect the disease activity [70]. Several case-control studies (microscopic colitis versus healthy control) have shown that fecal calprotectin levels were higher in patients with microscopic colitis than in patients with functional diarrhea [79,80,81]. 

Several studies have reported the utility of fecal biomarkers in microscopic colitis focused on both patients with active disease and patients in remission [79,81,82]. These three studies suggested the level of fecal calprotectin was significantly lower in patients with remission compared to those with active disease. Although most studies have recruited patients with active microscopic colitis, several papers have recruited patients with microscopic colitis in remission and measured the level of the fecal biomarker. Batista reported that the fecal calprotectin level is significantly decreased in patients in clinical remission compared to patients with active disease with a median of 436 μg/g (IQR 189–1300 μg/g) versus 56 μg/g (IQR 30–167 μg/g) (*p* = 0.004) [82]. Similar findings were found in a study which only included collagenous colitis patients and demonstrated that the median fecal calprotectin levels in active collagenous colitis at 80 μg/g compared with 26 μg/g in collagenous colitis in remission (*p* = 0.025) [59]. In the von Arnim study, fecal calprotectin levels were compared in patients with active microscopic colitis vs. microscopic colitis in remission and showed a significant elevation of fecal calprotectin in active microscopic colitis vs. microscopic colitis in remission vs. IBS (*p* < 0.0001); mean fecal calprotectin levels in active microscopic colitis, remission microscopic colitis, and IBS were 47.9 μg/g, 20.85 μg/g, and 1.9 μg/g, respectively [81].

### 7.3. Fecal Lactoferrin

Lactoferrin is an iron-binding protein in neutrophil granules [83]. A study done in 36 pediatric patients with IBD compared with 20 healthy controls demonstrated that a normal level of fecal lactoferrin can exclude intestinal inflammation with sensitivity, specificity, positive predictive value, and negative predictive value of 100%, 95%, 97.3%, and 100%, respectively [84]. Another study compared the level of fecal lactoferrin between IBS and IBD in 215 adult patients and reported that the sensitivity and specificity in distinguishing between the two diseases were 78% and 90% and suggested that an elevation of fecal lactoferrin was 100% specific in excluding IBS [85].

To date, only two studies have evaluated the utility of fecal lactoferrin in microscopic colitis. Fine reported that only three out of thirty-nine patients with microscopic colitis or celiac disease had a positive fecal lactoferrin test [86]. Wildt’s study included thirty-three patients with collagenous colitis, both active (n = 21) and in remission (n = 12); only one patient from the active collagenous colitis group had an increased fecal lactoferrin level. This particular patient had a negative fecal lactoferrin level during disease remission [82]. Fine studied the utility of fecal lactoferrin in differentiating patients with chronic diarrhea, including patients with microscopic colitis, celiac disease, enterocolitis, ulcerative colitis, and Crohn’s disease and demonstrated the presence of positive fecal lactoferrin in three of the thirty-nine microscopic colitis and celiac disease patients [86]. This study demonstrated the usefulness of fecal lactoferrin in IBD with a sensitivity and specificity of 90% and 98%, respectively. Burri reported a higher median level of fecal lactoferrin in patients with microscopic colitis at 6.2 μg/g (Q1–Q3: 3.4–118.9) compared to other patients with normal endoscopic findings at 1.0 μg/g (Q1–Q3: 1.0–1.0) [87]. 

In summary, fecal calprotectin and lactoferrin are not specific markers and can have increased levels in several disorders, including colorectal neoplasia, infectious diarrhea, upper gastrointestinal tract disease (Barrett’s esophagus, gastric ulcer, gastritis/duodenitis), gastric cancer, and history of non-steroidal anti-inflammatory drug use [76,87,88]. Therefore, clinicians should not rely on these biomarkers to establish a diagnosis and must integrate additional laboratory information into the clinical presentation.

### 7.4. Fecal Myeloperoxidase

Myeloperoxidase is a lysosomal protein found in neutrophils and is released into the phagosome of neutrophils during the degranulation process [89]. This enzyme can kill foreign microorganisms and damage normal tissue, leading to inflammation. Myeloperoxidase has been shown to be increased in IBD and has been studied in patients with microscopic colitis [90,91]. Wagner’s study reported a higher level of myeloperoxidase in patients with collagenous colitis and lymphocytic colitis than in healthy control patients with the level 10.4 μg/g (10–90 percentile: 0.39–24.5 μg/g), 9.6 μg/g (10–90 percentile: 0.39–15.6 μg/g), and 4.9 μg/g (10–90 percentile: 0.53–12.4 μg/g), respectively [90]. Lettesjö found an elevation of fecal myeloperoxidase in collagenous colitis (median 11.7 μg/g; Q1–Q3: 2.0–124 μg/g) compared to IBS patients (median 1.7 μg/g; Q1–Q3: 0.81–5.2 μg/g), *p* < 0.01, and healthy controls (median 2.5 μg/g; Q1–Q3:1.1–6.3 μg/g; *p* < 0.05 [91]. 

### 7.5. Fecal Eosinophils 

Eosinophils account for 1% to 4% of the total white blood cells in the body; however, eosinophils have a homeostatic role in immune and inflammatory responses [92]. During an inflammatory response, eosinophils may secrete inflammatory mediators that are stored in preformed vesicles. These eosinophilic granule proteins include eosinophilic cationic protein, major basic protein, eosinophil protein X, eosinophil-derived neurotoxin, and eosinophil peroxidase [92,93]. These proteins lead to tissue damage and increase toxic oxygen radicals [94]. Evidence of eosinophils in inflammatory bowel disease has been described; Lampinen demonstrated the numerous eosinophils in the inflamed intestine in a patient with ulcerative colitis and hypothesized that eosinophils lead to proinflammatory and promotility mechanisms that result in tissue destruction, stricture formation, and diarrhea in patients with ulcerative colitis [95]. Despite its important role in inflammatory responses, information about eosinophil protein in microscopic colitis is limited. Lettesjö studied the utility of fecal eosinophil protein X and demonstrated the increase in the level of eosinophil protein X in collagenous colitis (median 3.8 μg/g; Q1–Q3: 0.47–16.2 μg/g) compared to IBS (0.44 μg/g; Q1–Q3: 0.25–1.8 μg/g; *p* < 0.001) and healthy control patients (0.46 μg/g; Q1–Q3: 0.21–1.3 μg/g); *p* < 0.001) [91]. Wagner studied the fecal biomarkers in patients who presented with chronic non-bloody diarrhea, including nine patients with collagenous colitis, four patients with lymphocytic colitis, and forty-six with normal endoscopic findings [90]. The study revealed a higher level of fecal eosinophil protein X and fecal eosinophil cationic protein in collagenous colitis when compared with the normal diagnostic outcome with a *p*-value of 0.01. The mean fecal eosinophil cation protein levels in collagenous colitis and lymphocytic colitis were 5.3 μg/g (10–90 percentile: 0.8–13.8), 2.6 μg/g (10–90 percentile: 0.68–5.3) when compared with normal control 1.5 μg/g (0.42–2.8). The mean value of fecal eosinophil protein X in collagenous colitis and lymphocytic colitis was 5.7 μg/g (10–90 percentile: 0.06–12.3) and 1.7 μg/g (10–90 percentile: 0.43–3.9) when compared with normal control 0.82 μg/g (10–90 percentile: 0.12–1.9) [90]. This study suggested fecal eosinophil protein X and fecal eosinophil cationic protein can be used to predict the diagnostic outcome of collagenous colitis [90]. In one study, the colonic mucosa in patients with collagenous colitis was infiltrated with activated eosinophils [96]. However, there are limitations since the paper focused only on patients with collagenous colitis.

### 7.6. Fecal Mast Cells

Mast cells have been shown to have an association with multiple gastrointestinal tract diseases, including chronic diarrhea of unknown etiology with normal colonoscopy [97], diarrhea-predominant irritable bowel syndrome [98], and IBD [93]. Chi studied the pathology tissue of 64 patients, consisting of 29 patients with collagenous colitis, 35 patients with lymphocytic colitis, and 20 control patients [99]. There was a higher number of mast cells in the lamina propria of patients with collagenous colitis (mean highest mast cell count 39/high power field (HPF) and lymphocytic colitis (30/HPF) compared to the control (23/HPF) with a *p*-value < 0.01 [99]. The patients with collagenous colitis had increased tryptase levels compared to the control patients, 40% compared to 10%, respectively, with a *p*-value of <0.01 [99]. This study has limitations in tissue location, and analysis of the location was not performed. 

## 8. Management

This section will provide a brief overview of the treatment of patients with microscopic colitis. In particular, the two most useful drugs provide some insight into the pathogenesis of this disorder. The European guidelines recommend the removal of drugs that can precipitate microscopic colitis in any patient with a new diagnosis of microscopic colitis [1]. These drugs include proton pump inhibitors, nonsteroidal anti-inflammatory drugs, and tobacco. The initial treatment should start with budesonide at 9 mg daily for 6 to 8 weeks. Patients on this drug have a clinical response, a histologic response, and improved quality of life. The fact that this drug is a corticosteroid medication would suggest that its primary effect is on either inflammation in colonic mucosa or an immune-mediated disease in the colonic mucosa. These guidelines also recommend the use of bile acid binding agents in patients with bile acid diarrhea and microscopic colitis. However, the studies summarized in earlier paragraphs indicate that these medications can also be useful in patients who do not have laboratory evidence of bile acid malabsorption. The exact effect of reducing the bile acid load in these patients is uncertain but does establish a potential role in the pathogenesis of the diarrhea in these patients. These guidelines recommend against the use of mesalazine, probiotics, methotrexate, and prednisolone. The guidelines indicate there is inadequate evidence to make a recommendation regarding the use of bismuth subsalicylate, loperamide, and antibiotics. Decisions regarding the use of biological drugs and surgery should be limited to experts in the management of these patients.

## 9. Conclusions

Microscopic colitis causes chronic diarrhea, which can have profound effects on the quality of life of the individuals with this diagnosis. It occurs more frequently in older women but can be associated with autoimmune disorders which need consideration during the initial evaluation of these patients. The pathogenesis may involve an underlying genetic disorder, changes in the colonic microbiome, immune-related colonic injury, and/or bile acid malabsorption. In most patients, the mucosa appears normal during colonoscopy. Biopsies taken either in the right or left colon reveal either collagenous or lymphocytic colitis. Stool studies are essential to rule out alternative causes of diarrhea but cannot establish this diagnosis. Treatment usually starts with oral budesonide. This drug improves both clinical symptoms and histology. Some patients with and without bile acid malabsorption respond to bile acid sequestrants. Bile acid sequestrants might provide a useful alternative in patients who do not respond to budesonide or cannot use budesonide for sustained periods. In summary, microscopic colitis is an important consideration in patients with chronic unexplained diarrhea and has relatively good treatment options. 

## Data Availability

Not applicable.

## Abbreviations

CD: cluster of differentiation; CD4^+^, helper T cell; CD8^+^, cytotoxic T cell; FXR, Farnesoid X-receptor; HLA, human leukocyte antigen; HPF, high power field; IBD, inflammatory bowel disease; IBS, irritable bowel syndrome; IgG, immunoglobulin G; IQR, interquartile range; OR, odds ratio; ^75^SeHCAT, ^75^Selenium-HomoCholic Acid Taurine; SNP, single nucleotide polymorphism.
